# Plant-Derived miRNAs as Potential Cross-Kingdom Cancer Regulators

**DOI:** 10.3390/genes16121441

**Published:** 2025-12-02

**Authors:** Aizhan Rakhmetullina, Zuzanna Lubas, Piotr Zielenkiewicz

**Affiliations:** 1 Institute of Biochemistry and Biophysics, Polish Academy of Sciences, ul. Pawińskiego 5a, 02-106 Warsaw, Poland; arakhmet@ibb.waw.pl (A.R.); zlubas@ibb.waw.pl (Z.L.); 2 Institute of Experimental Plant Biology and Biotechnology, University of Warsaw, ul. Miecznikowa 1, 02-096 Warsaw, Poland

**Keywords:** plant-derived miRNAs, cross-kingdom regulation, cancer therapeutics, dietary miRNAs, gene expression regulation, tumour suppressors, oncogenes

## Abstract

MicroRNAs (miRNAs) are key posttranscriptional regulators of gene expression that influence cancer initiation, progression, and therapeutic response. While most studies have focused on endogenous miRNAs, emerging evidence has highlighted the role of plant-derived miRNAs as exogenous dietary regulators capable of cross-kingdom gene modulation. This review summarises current knowledge regarding plant-derived miRNAs and their ability to regulate human cancer-related genes. Experimental findings indicate that plant miRNAs can withstand gastrointestinal digestion, enter the circulation, and regulate the expression of oncogenes, tumour suppressors, long noncoding RNAs, and immune checkpoint molecules via canonical RNA-induced silencing mechanisms. Specific examples include miR-156a, miR-159a-3p, miR-166a, miR-167e-5p, miR-171, miR-395e, miR-2911, miR-4995 and miR-5754, which exhibit anticancer activities across various cancer types and modulate key signalling pathways in mammalian cells, highlighting their potential as cross-kingdom regulators with therapeutic relevance. In addition to these characterised miRNAs, certain plant groups, which are rich in bioactive compounds, remain unexplored as sources of functional miRNAs, representing a promising avenue for future research. Collectively, these studies underscore the ability of plant-derived miRNAs to modulate mammalian gene expression and suggest their potential as diet-based or synthetic therapeutic agents. Further investigations into their bioavailability, target specificity, and functional relevance could inform innovative strategies for cancer prevention, integrating nutritional, molecular biological, and therapeutic approaches.

## 1. Introduction

MicroRNAs (miRNAs) are important modulators of gene expression that function as oncogenes or tumour suppressors related to various cancers [1,2]. Although the majority of studies have concentrated on endogenous miRNAs, new data emphasise the function of dietary plant-derived miRNAs as exogenous regulators that may have therapeutic value [3,4,5]. The influence of different diets on human health has been studied for many years [6,7]. Diet has long been linked to disease prevention, with plant-based foods providing bioactive compounds such as polyphenols [8,9], alkaloids [10,11], and saponins [12,13]. Scientific studies have demonstrated that plant-based foods function not only as sources of energy and nutrients but also as carriers of bioactive compounds capable of modulating human physiological processes [14]. The discovery that plant-derived miRNAs may be involved in these health impacts adds a novel molecular facet to the diet–cancer relationship [15].

Plant-derived miRNAs have been identified as novel diet-related molecular regulators with the potential to affect human health via cross-kingdom gene regulation [16,17]. miRNAs are short (approximately 22 nucleotides in length), noncoding RNA molecules that regulate gene expression [18,19]. In plants, they play key roles in growth [20], biotic and abiotic stress responses [21,22], and development [23,24]. While both plant and animal miRNAs regulate gene expression, they differ in their sequences [25,26], biogenesis [25,27], and mechanisms by which they perform similar functions [26,27]. In plants, the miRNA duplex is modified by the methyltransferase HEN1 through 2′-O-methylation at the 3′ end, a process that enhances its stability compared to animal miRNAs [28,29]. Recent studies have suggested that once ingested through the diet, plant-derived miRNAs may survive digestion in the gastrointestinal tract [30,31], enter the bloodstream [30,32], and regulate the expression of endogenous mRNAs [17,30]. They have been detected in different mammalian biological fluids [33] and tissues, including blood [3,4], milk [34,35,36], urine [4] and various organs [37,38,39], highlighting their potential to reach the systemic circulation [3,4,40] and modulate host cellular processes. Zhang et al. provided early evidence that the plant-derived miRNA miR168a may be found in the serum and plasma of both humans and animals after they consume rice [41]. In a related study, Liang et al. reported that exogenous plant miRNAs, such as miR-172 from *Brassica oleracea (B. oleracea)*, can survive gastrointestinal digestion and be detected in the stomachs, intestines, serum, faeces, and multiple organs, including the spleen, liver, and kidneys, of mice, indicating that dietary miRNAs can reach the circulation and potentially interact with host cells [42]. Consistent with these findings, plant miRNAs, including miR166a, miR156a, miR157a, miR172a, and miR168a, have also been identified in human breast milk, suggesting that dietary miRNAs may be transmitted to infants and contribute to early development [34]. Interestingly, cross-kingdom regulation has been observed in other mammals. In 2025, Tan et al. investigated the uptake of plant-derived miRNAs from bamboo in giant pandas. They identified 57 bamboo-derived miRNAs in the peripheral blood of both juvenile and adult pandas and demonstrated through functional analyses that these miRNAs can regulate genes involved in dopamine metabolism, suggesting their role in shaping dietary preferences [43]. An alternate strategy involves the synthesis of plant-derived miRNAs, which can subsequently be applied in medical applications.

This review focuses on plant-derived miRNAs and their interactions with human cancer-related genes and summarises recent experimental evidence in this emerging field.

To collect data on plant-derived microRNAs associated with cancer treatment, we performed a targeted literature search in PubMed using the following query: (“cancer” OR “carcinoma” OR “tumour” OR “neoplasm”) AND (“plant microRNA” OR “plant-derived miRNA” OR “exogenous miRNA”) AND (“in vitro” OR “cell line” OR cells OR “cell culture”). From the retrieved results, we included only those studies that provided experimental evidence demonstrating the ability of plant-derived miRNAs to regulate cancer-related processes. The identified studies were systematically reviewed, highlighting the specific plant miRNAs examined, their molecular targets, the experimental models used, and the reported effects on cancer progression and tumour-related pathways. We also examine the conservation of certain miRNAs across multiple plant species, which suggests that some plant-derived miRNAs may exert broad-spectrum regulatory effects in mammalian systems. Furthermore, we summarize the available evidence on the anticancer effects of berries, which are rich in bioactive compounds and long used in both traditional and modern medicine for the cancer prevention and treatment but remain underexplored as potential sources of plant-derived miRNAs. For berries, we focused on publications from 2015–2025, using a detailed PubMed query targeting relevant species and experimental studies, while excluding in silico works.

## 2. Plant-Derived miRNAs and Cancer

The role of miRNAs in cancer is well established, as they modulate the expression of oncogenes and tumour suppressor genes, thereby influencing cancer initiation, progression, and therapeutic response [44,45]. Emerging evidence has indicated that plant-derived miRNAs obtained through dietary intake can enter mammalian cells and modulate gene expression, representing a novel cross-kingdom mechanism with therapeutic potential [3,46,47].

Plant miRNAs can enter the human body primarily through the gastrointestinal tract [48,49]. Following ingestion, these miRNAs are transported in the bloodstream either in combination with proteins or encapsulated within extracellular vesicles (EVs) such as exosomes and microvesicles [50,51,52]. Recent studies have shown that miRNAs can be encapsulated within exosomes [52,53], which protect them from harsh gastrointestinal conditions such as digestive enzymes [54] and acidic pH [55]. This enables their transfer to neighbouring or distant cells to modulate cellular functions. These stable miRNAs have been detected in a wide range of body fluids, including serum, milk, saliva, and urine [56,57,58]. In one of the studies carried out by our group [34], publicly available miRNA sequencing data from mammalian breast milk exosomes were analysed, and qRT-PCR experiments were performed to assess the presence of five plant-derived miRNAs (miR-166a, miR-156a, miR-157a, miR-172a, and miR-168a) in breast milk from healthy volunteers. All five miRNAs were detected in whole milk, while only two were confirmed in exosomal fractions, with concentrations ranging from 4 to 700 fM.

Dietary plant miRNAs can enter mammalian cells and bind to complementary sequences within the 3′ untranslated regions (3′ UTRs) of target mRNAs, leading to mRNA degradation or translational repression [59]. This gene-silencing activity is mediated by the RNA-induced silencing complex (RISC), which incorporates exogenous miRNA and guides it to its specific target, enabling precise posttranscriptional regulation. While dietary uptake represents a natural route for cross-kingdom regulation, advances in biotechnology now allow the synthesis of plant-derived miRNAs. These synthetic molecules can be generated and applied in preclinical models to assess their functional roles and therapeutic potential. Among the plants investigated for their therapeutic potential, *Moringa oleifera*, widely used in African traditional medicine, has recently attracted attention because its microvesicles contain miRNAs that influence tumour cell proliferation and apoptosis [60].

To present a comprehensive overview of plant-derived miRNAs with experimentally validated roles in regulating cancer-related gene expression, we summarise key studies in Table 1. These findings emphasise the diversity of plant miRNAs, their specific molecular targets, and the cancer types in which they demonstrate functional activity. Several plant miRNAs have shown promise in preclinical studies because of their anticancer properties. For instance, oral administration of a plant-derived miR-159 mimic substantially reduced xenograft breast tumour growth in mice. Derived from *Arabidopsis thaliana* (*A. thaliana*), miR-159 has been demonstrated to suppress the Wnt signalling pathway by targeting *TCF7*, leading to decreased MYC protein expression and inhibition of breast cancer cell proliferation. These findings provide the first direct evidence that a plant-derived miRNA can modulate cancer growth in mammals, highlighting its potential as a therapeutic agent [61].

Similarly, soybean-derived miR-159a-3p demonstrated anticancer effects in human colon carcinoma (Caco-2) cells, where it suppressed proliferation and induced apoptosis without affecting normal mucosal cells. The gma-miR-159a mimic reduced TCF7 protein expression, resulting in decreased cell proliferation and highlighting the ability of plant-derived miRNAs to regulate oncogenic pathways across different tissue types [65]. This cross-tissue regulatory potential is further illustrated by miR-2911 from *Lonicera japonica* (*L. japonica*), which has been shown to target *TGFβ1* in colon cancer cells, leading to inhibition of tumour growth [68]. The authors demonstrated that miR-2911 binds to the 3′-UTR of *TGF-β1* mRNA, resulting in decreased protein expression. Importantly, compared with other honeysuckle-derived miRNAs, miR-2911 is remarkably stable during the boiling process, highlighting its potential as a plant-derived therapeutic agent and shedding light on the development of orally deliverable small RNA therapeutics [73]. In HPV-associated cervical cancer, the same miRNA directly targets the viral oncogenes *E6* and *E7*, resulting in their reduced expression, inhibition of cell proliferation, and induction of apoptosis in HPV-positive cell lines. Notably, both synthetic miR-2911 and miR-2911-containing exosomes produced these effects, indicating that miR2911 can potentially be used for the development of novel therapeutic strategies in HPV-associated cervical cancers [71]. These findings highlight the functional diversity of individual plant miRNAs in modulating diverse cancer-related pathways, depending on the tissue context and molecular targets. Another example of a tissue-specific yet multifunctional plant miRNA is miR-166a from *Lycium barbarum* (*L. barbarum*). In kidney cancer, miR-166a downregulated the expression of multiple cell cycle regulators, including *CCNA2*, *CCND1*, *CCNE2*, *CCNB1*, and *CDC20*, effectively inhibiting proliferation [69]. The same miRNA, lb-miR-166a, was shown to directly target *STK39* in triple-negative breast cancer cells, suppressing proliferation, invasion, and metastasis while promoting apoptosis through the inhibition of the STK39/MAPK14 signalling pathway [70].

In animal cells, gene expression is typically controlled by endogenous miRNAs through interactions with the 3′ UTRs of target genes, and exogenous plant miRNAs can act through the same mechanism. For example, a broccoli-derived miR156a mimic inhibited epithelial–mesenchymal transition in nasopharyngeal cancer cells by directly targeting the 3′ UTR of Junctional adhesion molecule A (*JAMA*), a gene involved in cell adhesion and migration [62]. This example further illustrates that plant-derived miRNAs can regulate mammalian gene expression through the same posttranscriptional mechanisms as endogenous miRNAs can, thereby modulating key processes in cancer progression.

**Figure 1:**
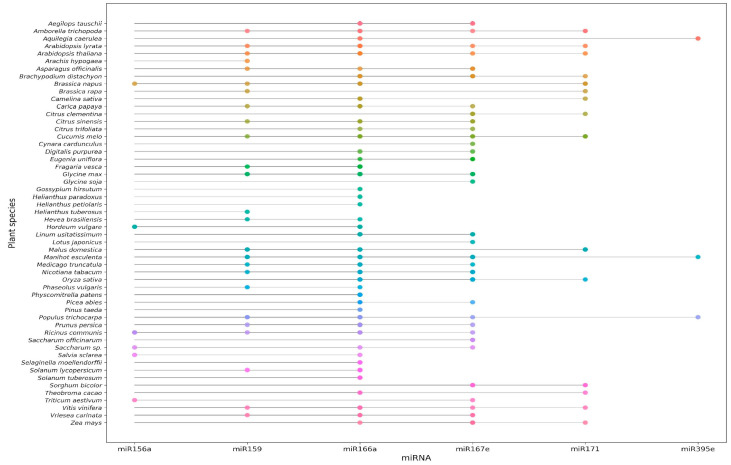
Conserved plant miRNA sequences across species. Note: Selected plant miRNAs display high sequence conservation across multiple species, highlighting their potential for broad regulatory functions. Examples include the following: miR-156a (UGACAGAAGAGAGUGAGCACA) is conserved across 6 plant species. miR-159a-3p (UUUGGAUUGAAGGGAGCUCUA) is conserved across 25 plant species. miR-166a (UCGGACCAGGCUUCAUUCCCC) is conserved across 44 plant species. miR-167e-5p (UGAAGCUGCCAGCAUGAUCUG) is conserved across 33 plant species. miR-171 (UGAUUGAGCCGCGCCAAUAUC) is conserved across 15 plant species. miR-395e (CUGAAGGGUUUGGAGGAACUC) is conserved across 3 plant species.

Some plant-derived miRNAs are highly conserved across multiple species, suggesting the potential for broad-spectrum regulatory activity in mammalian systems and making them promising candidates for further functional studies. To assess sequence conservation, we retrieved 10,414 miRNA sequences from 86 plant species in miRBase v22 (http://www.mirbase.org/, accessed on 13 September 2025) and analysed identical sequences across species. This analysis enabled us to highlight six conserved anticancer plant miRNAs and the plant species in which they are present. One such miRNA, miR-167-5p, is highly conserved and abundantly expressed across the plant kingdom. According to miRBase (https://www.mirbase.org/), this miRNA sequence (UGAAGCUGCCAGCAUGAUCUA) is shared by 33 plant species (Figure 1), including rice [74,75], wheat [76], and maize [77], which collectively constitute a major part of the human and animal diet. miR-167e-5p suppresses intestinal cell proliferation by directly targeting β-catenin, a central component of the Wnt signalling pathway, in both porcine jejunum epithelial cells (IPEC-J2) and Caco-2 cells [64].

These conserved miRNAs may serve as candidates for cross-kingdom functional studies. For instance, miR-171, which is conserved across 15 plant species, has been reported to have tumour-suppressive effects. The conservation of miR-171 across these species suggests that its regulatory function may be preserved across diverse plants, making it crucial determine whether plant-derived miR-171 can produce biological effects in humans. Gismondi et al. demonstrated that a synthetic miR-171 mimic significantly reduced *GNA12* expression at both the mRNA and protein levels, as evidenced through qPCR and Western blot analyses [67]. This effect was mediated through direct targeting of *GNA12*, a member of the G protein family that plays a central role in cancer progression and metastasis in oral, breast, and prostate adenocarcinoma cell lines. These examples demonstrate that highly conserved plant-derived miRNAs can modulate key signalling pathways in mammalian cells, demonstrating their potential as cross-kingdom regulators with therapeutic relevance.

Extending these observations, plant-derived miRNAs have also been shown to regulate noncoding RNAs in human cancer cells. Marzano et al. demonstrated that the plant-derived miRNAs mtr-miR-5754 from *Medicago truncatula* (*M. truncatula*) and gma-miR-4995 from *Glycine max* (*G. max*) directly target the long noncoding RNAs *MALAT1* and *NEAT1*, respectively, in HCT116 colon carcinoma cells [66]. Transfection of these miRNA mimics led to a significant reduction in the expression levels of *MALAT1* and *NEAT1*, as confirmed by RT–qPCR. Luciferase reporter assays validated the sequence-specific binding of these miRNAs to their target sites within *MALAT1* and *NEAT1*. These results highlight the ability of plant-derived miRNAs to modulate both protein-coding and noncoding RNAs in mammals, suggesting new avenues for biotechnological and therapeutic applications.

Plant-derived miRNAs have also been shown to target immune-related pathways. A recent study investigated the effects of mes-miR-395e, a plant-derived miRNA from *Manihot esculenta* (cassava), on *PD-L1* expression in renal cell carcinoma (RCC) cell lines (786-O and KIJ265T) derived from primary tumours. Researchers transfected synthetic mes-miR-395e mimics into these cells, and subsequent analyses revealed a significant reduction in *PD-L1* expression at both the mRNA and protein levels. Luciferase reporter assays indicated that mes-miR-395e does not directly bind to *PD-L1* mRNA, suggesting an indirect regulatory effect. These findings highlight the potential of plant-derived miRNAs, such as mes-miR-395e, to modulate immune checkpoint proteins such as *PD-L1*, offering a novel approach to cancer immunotherapy [72]. Overall, these findings highlight the ability of exogenous plant miRNAs to modulate target gene expression and suggest their potential for therapeutic applications in humans.

Although the studies discussed above demonstrate that dietary plant-derived miRNAs may withstand digestion, enter the bloodstream, and modulate host gene expression, their diversity and functional relevance across different plant species remain largely unexplored. This emerging perspective aligns with the long-standing role of traditional plant-based medicine in human health, in which natural bioactive compounds such as polyphenols [78,79], saponins [80], alkaloids [81], and tannins [82,83] have attracted considerable interest because of their therapeutic potential and relatively low risk of side effects. Among the various sources of medicinal plants, berry fruits represent a notable group of species characterised by their high abundance of bioactive compounds and have long been used in both traditional and modern medicine for the prevention and treatment of cancer [84].

**Figure 2:**
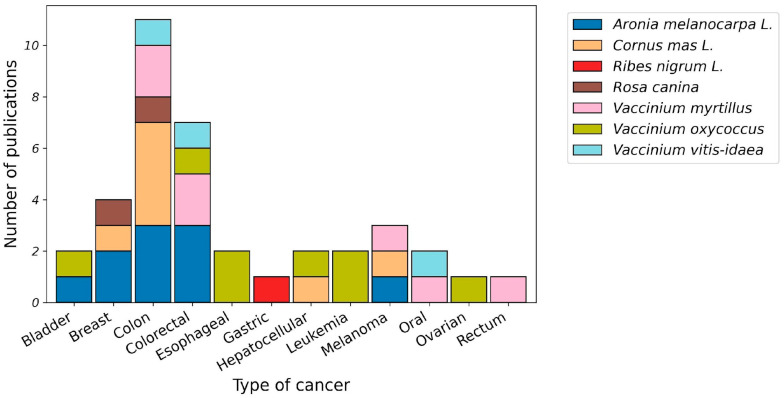
Berries and their anticancer effects: experimental studies from 2015–2025.

In Figure 2, we highlight the available evidence on the anticancer effects of berries, which, despite their potential, remain underexplored as sources of plant-derived miRNAs.

The data illustrate the number of experimental publications investigating the anticancer effects of various berries across different cancer types over the period from 2015 to 2025. Colorectal, colon, and breast cancers appear to be the most frequently studied, with the greatest number of publications, particularly for *Aronia melanocarpa* L. (*A. melanocarpa* L.), *Cornus mas* L. (*C. mas* L.), and *Vaccinium myrtillus* (*V. myrtillus*). Studies related to their roles in other cancers, such as gastric, hepatic, and ovarian cancers, are rare, indicating that these areas remain underexplored. Each colour in the bars represents a specific berry species, highlighting the contribution of individual berries to research efforts for each cancer type. Overall, the data emphasise that while berries have been widely investigated for certain cancers, significant gaps exist in the experimental evaluation of their anticancer effects across other tumour types. These findings also underscore the potential for future studies, particularly regarding plant-derived miRNAs, to elucidate novel molecular mechanisms underlying these effects.

Notably, preclinical studies have shown that certain berries have significant anticancer effects. For instance, anthocyanin-rich black currant (*Ribes nigrum* L.) extract exhibited protective effects against hepatocellular carcinoma in rats, significantly reducing the numbers, sizes, and volumes of preneoplastic hepatic nodules. Histopathological analysis confirmed decreased tumour development, whereas immunohistochemical evaluation revealed reduced cell proliferation and increased apoptosis. These findings suggest that black currant anthocyanins may protect against liver carcinogenesis and have potential as preventive agents [85]. Similarly, black chokeberry (*A. melanocarpa* L.) fruit peel extracts have been shown to exert anticancer effects in preclinical models of breast carcinoma. Both in vitro and in vivo studies have demonstrated antiproliferative activity, induction of apoptosis, and modulation of mitochondrial function while also enhancing the responsiveness of cancer cells to chemotherapy [86]. Cornelian cherry (*C. mas* L.) fruit extracts also showed anticancer activity, reducing the viability of MCF-7 human breast cancer cells in a dose- and time-dependent manner. Treatment with the extract significantly induced apoptosis, an effect attributed to its high phenolic and flavonoid contents and antioxidant properties [87]. Likewise, rose hip (*Rosa canina* L.) extracts have demonstrated antiproliferative and pro-apoptotic effects against human cancer cells, including lung and prostate cancer lines, supporting their therapeutic potential as a source of bioactive compounds for cancer prevention and therapy [88]. In addition, black nightshade (*Solanum nigrum* Linn.) has shown promising anticancer activity against digestive system tumours. Compounds such as solanine, solasonine, and solamargine induce apoptosis, cause cell cycle arrest, inhibit metastasis, and increase sensitivity to chemotherapy. These results support the potential of *S. nigrum* as a therapeutic agent for digestive system cancers [89]. Lingonberry (*Vaccinium vitis-idaea*) and bilberry (*V. myrtillus*) have also demonstrated anticancer properties, particularly against digestive tract tumours. In animal models, bilberry extracts reduced the number of colonic aberrant crypt foci, whereas lingonberry supplementation reduced adenoma growth, suggesting a protective effect through antiproliferative and pro-apoptotic mechanisms [90]. Cranberries (*Vaccinium macrocarpon*) are rich in ursolic acid and its esters, which have been shown to inhibit the proliferation of DU145 prostate cancer cells and suppress the activities of matrix metalloproteinases (MMP-2 and MMP-9) and enzymes involved in tumour invasion and metastasis, highlighting their chemopreventive and antimetastatic potential [91]. Taken together, these studies highlight the diverse anticancer effects of bioactive compounds. Nevertheless, the underlying mechanisms through which such herbal medicines affect cancer remain poorly understood, and the potential role of berry-derived miRNAs is still unexplored. Investigating whether these fruits contain miRNAs with functional relevance in cancer biology could provide a novel molecular perspective on their health-promoting properties and advance the development of new plant-based therapeutic strategies.

Despite promising results, several limitations apply to the therapeutic application of plant-derived miRNAs. Many studies lack strict dietary controls and frequently use mixed plant extracts, which complicates the interpretation of results and makes it difficult to attribute observed effects to specific miRNAs. Efficient and safe delivery systems are also critical to ensure that miRNAs reach target tissues or cells while minimizing off-target effects. In this context, EVs [92] have emerged as promising natural carriers that enhance miRNA stability and facilitate targeted delivery. Both EV encapsulation and synthetic nanocarriers [92,93] can protect plant-derived miRNAs from enzymatic degradation, improve biodistribution, and increase therapeutic efficacy through more efficient cellular uptake. Addressing these challenges is essential to advance dietary miRNAs toward reliable therapeutic applications.

## 3. Future Perspectives

Although plant-derived miRNAs have emerged as potential cross-kingdom regulators, their roles in human health and disease remain largely unexplored. While the therapeutic properties of numerous medicinal plants have been studied, the contribution of plant miRNAs is still in its early stages. Berries represent a promising yet underinvestigated group, with miRNAs identified from only a few species, such as *R. nigrum*, to date [94]. Two, rni-miR-5021 and rni-miR-5185, were discovered for the first time, and their presence and expression were confirmed in vivo using standard molecular techniques. Characterising berry-derived miRNAs and their functional relevance could provide novel molecular insights and open new avenues for plant-based therapeutic strategies. We hope to address this knowledge gap in the near future through further research on berry-derived miRNAs and their potential biological effects.

Comprehensive characterisation and functional analysis of berry-derived miRNAs may provide novel insights into their bioactivity and help elucidate new molecular pathways through which they contribute to human health. Such insights could help explain some of the therapeutic effects long attributed to berries in both traditional and modern medicine. Moreover, understanding the diversity and functional relevance of these miRNAs across species will not only expand our knowledge of plant–human interactions but also lay the groundwork for innovative applications, ranging from dietary interventions and functional foods to the development of novel therapeutic strategies based on plant-derived miRNAs. This requires further studies to characterize the concentrations of plant-derived miRNAs in human tissues.

## 4. Conclusions

Plant-derived miRNAs represent an emerging class of diet-related molecular regulators capable of modulating cancer-related pathways across species. Experimental evidence has demonstrated that exogenous plant miRNAs can survive digestion, enter the circulation, and regulate mammalian gene expression, thereby influencing oncogenes, tumour suppressors, noncoding RNAs, and immune checkpoint molecules. These findings not only underscore the concept of diet as a source of functional regulators beyond classic phytochemicals but also highlight the therapeutic potential of exogenous small RNAs.

The mechanisms underlying the uptake, stability, and bioavailability of dietary miRNAs remain incompletely understood. Moreover, the potential contribution of plant-derived miRNAs from underexplored plants, such as berries, is largely unknown. Systematic identification and functional validation of these molecules could reveal new therapeutic strategies.

Taken together, the recent evidence highlights plant-derived miRNAs as promising molecular regulators of the interplay between nutrition and cancer biology. Expanding this research will be critical for clarifying their role in cancer prevention and treatment and for applying these insights to clinically relevant applications, including dietary interventions and RNA-based therapeutics.

## Figures and Tables

**Table 1:** Plant-derived miRNAs and their reported anticancer implications.

No.	miRNA	Plant	Target	In Vitro/ In Vivo	Cell Line/Animal Model	Type of Cancer	Year	Ref.
1	miR-159	*A.thaliana*	*TCF7*	in vitro/in vivo	MDA-MB-231 MCF-10A/NSG mice	breast	2016	[61]
2	miR-156a	*B.oleracea*	*JAMA*	in vitro	CNE2, HONE1, C666-1	nasopharyngeal	2016	[62]
3	miR-167a	*B.oleracea*	*IRS1*	in vitro	PANC-1, HEK293T	pancreatic adenocarcinoma	2023	[63]
4	miR-167e-5p	33 plants *	*β-catenin*	in vitro	IPEC-J2, Caco-2	colorectal	2019	[64]
5	miR-159a-3p	*Glycine Max*	*TCF7*	in vitro	NCM460, Caco-2	colon	2020	[65]
6	miR-5754	*Medicago truncatula*	*MALAT1*	in vitro	LAN-1, T98G, HepG2, HCT116	colon	2020	[66]
7	miR-4995	*Glycine Max*	*NEAT1*	in vitro	LAN-1, T98G, HepG2, HCT116	colon	2020	[66]
8	miR-171	15 plants **	*GNA12*	in vitro	HEK293	oral, breast, prostate adenocarcinoma	2021	[67]
9	miR-2911	*Lonicera japonica*	*TGFβ1*	in vitro/in vivo	CT26/C57Bl/6, BALB/c mice	colon	2021	[68]
10	miR-166a	*Lycium barbarum*	*CCNA2*, *CCND1*, *CCNE2*, *CCNB1*, *CDC20*	in vitro/in vivo	Caki-1, ACHN/nude mice	kidney	2023	[69]
11	miR-166a	*Lycium barbarum*	*STK39*	in vivo/in vitro	MB-231/nude mice	triple-negative breast cancer	2024	[70]
12	miR-2911	*Lonicera japonica*	*E6/E7*	in vitro	HEK293T, CaSki, SiHa, HeLa	cervical	2024	[71]
13	miR-395e	*Manihot esculenta*	*PD-L1*	in vitro	RCC 786-O, Caki-1, KIJ265T	renal	2025	[72]

Note: miR167e-5p (33 plants) * and miR171 (15 plants) ** are conserved across multiple plant species; exact sources are not specified. ACHN—human renal adenocarcinoma cell line; C666-1—human nasopharyngeal carcinoma cell line; Caco-2—human colon adenocarcinoma cell line; Caki-1—human renal cell carcinoma; CaSki—HPV16-positive human cervical cancer cell line; CNE2—human nasopharyngeal carcinoma cell line; CT26—mouse colorectal adenocarcinoma cells; HCT116 p53+/+—human colorectal carcinoma cell line, p53 wild-type; HCT116 p53−/−—isogenic HCT116 cell line engineered to lack p53; HEK293—human embryonic kidney cells; HeLa—HPV18-positive human cervical cancer cell line; HepG2—human liver cancer cell line; HONE1—human recurrent nasopharyngeal carcinoma cell line; IPEC-J2—non-transformed human jejunal epithelial cell line; KIJ265T—human renal carcinoma cell line; LAN-1-p53−/−—human neuroblastoma cell line lacking functional p53; MCF-10A—non-cancerous human mammary epithelial cell line; MDA-MB-231—human triple-negative breast cancer cell line, metastatic mammary adenocarcinoma; NCM460—human normal colonic mucosal epithelial cell line; PANC-1—Pancreatic carcinoma-1; RCC 786-O—human renal cell carcinoma; SiHa—HPV16-positive human cervical cancer cell line; T98G—human glioblastoma cell line.

## Data Availability

All relevant data are presented within the paper.
